# Tollip, an early regulator of the acute inflammatory response in the substantia nigra

**DOI:** 10.1186/s12974-016-0766-5

**Published:** 2016-12-07

**Authors:** Marie Humbert-Claude, D. Duc, D. Dwir, L. Thieren, J. Sandström von Tobel, C. Begka, F. Legueux, D. Velin, M. H. Maillard, K. Q. Do, F. Monnet-Tschudi, L. Tenenbaum

**Affiliations:** 1 Laboratory of Cellular and Molecular Neurotherapies, Center for Neuroscience Research, Department of Clinical Neuroscience, Lausanne University Hospital, Lausanne, Switzerland; 2 Department of Psychiatry, Center for Psychiatric Neuroscience, Centre Hospitalier Universitaire Vaudois, University of Lausanne, Prilly, Lausanne, Switzerland; 3 Department of Physiology, University of Lausanne, Lausanne, Switzerland; 4 Service of Gastroenterology and Hepatology, Department of Medicine, Lausanne University Hospital, Lausanne, Switzerland; 5 FIRALIS SA, Huningue, France

**Keywords:** Tollip, Substantia nigra, Lipopolysaccharide, Neuroinflammation, iNOS, Oxidative stress, Cytokine, Adeno-associated viral vector, Parkinson’s disease, Toll-like interacting protein

## Abstract

**Background:**

Tollip is a ubiquitously expressed protein, originally described as a modulator of the IL-1R/TLR-NF-κB signaling pathways. Although this property has been well characterized in peripheral cells, and despite some evidence of its expression in the central nervous system, the role of Tollip in neuroinflammation remains poorly understood. The present study sought to explore the implication of Tollip in inflammation in the substantia nigra pars compacta, the structure affected in Parkinson’s disease.

**Methods:**

We first investigated Tollip distribution in the midbrain by immunohistochemistry. Then, we addressed TLR4-mediated response by intra-nigral injections of lipopolysaccharide (LPS), a TLR4 agonist, on inflammatory markers in Tollip knockout (KO) and wild-type (WT) mice.

**Results:**

We report an unexpectedly high Tollip immunostaining in dopaminergic neurons of the mice brain. Second, intra-nigral injection of LPS led to increased susceptibility to neuroinflammation in Tollip KO compared to Tollip WT mice. This was demonstrated by a significant increase of tumor necrosis factor alpha (TNF-α), interleukin 1 beta (IL-1β), interleukin 6 (IL-6), and interferon gamma (IFN-γ) messenger RNA (mRNA) in the midbrain of Tollip KO mice upon LPS injection. Consistently, brain rAAV viral vector transduction with a nuclear factor kappa B (NF-κB)-inducible reporter gene confirmed increased NF-κB activation in Tollip KO mice. Lastly, Tollip KO mice displayed higher inducible NO synthase (iNOS) production, both at the messenger and protein level when compared to LPS-injected WT mice. Tollip deletion also aggravated LPS-induced oxidative and nitrosative damages, as indicated by an increase of 8-oxo-2′-deoxyguanosine and nitrotyrosine immunostaining, respectively.

**Conclusions:**

Altogether, these findings highlight a critical role of Tollip in the early phase of TLR4-mediated neuroinflammation. As brain inflammation is known to contribute to Parkinson’s disease, Tollip may be a potential target for neuroprotection.

**Electronic supplementary material:**

The online version of this article (doi:10.1186/s12974-016-0766-5) contains supplementary material, which is available to authorized users.

## Background

Neuroinflammation is thought to be an important contributor to the pathogenesis of Parkinson’s disease (PD) [1, 2]. Indeed, the presence of pro-inflammatory cytokines in postmortem brain samples from PD patients [3], microglial activation evidenced in patients brain by PET scan [4], alteration in the composition of lymphocyte populations in patients’ blood [5], and activation of the innate immune surveillance system [6] as well as a genetic association with the HLA-DRA locus (coding for major histocompatibility complex II molecules) [7, 8] strongly support this hypothesis. Chronic inflammation is thought to cause the degeneration of dopaminergic neurons [9, 10] as shown by findings in toxin-induced [11] and genetic models of PD [12, 13]. Interestingly, the emerging concept of a decisive role of microglia priming in the pathogenesis of neurodegenerative diseases supports a role for acute inflammation in neurodegeneration [14, 15].

Enzymes associated with oxidative (i.e., COX-1 and COX-2) and nitrosative stresses (i.e., iNOS) have also been involved in neuronal vulnerability in PD [16, 17]. Furthermore, due to their high iron content [18] and reduced glutathione levels [19], dopaminergic neurons are thought to be particularly susceptible to reactive oxygen species released by activated microglia.

Lipopolysaccharides (LPS), endotoxins found in the outer membrane of gram-negative bacteria, were suggested to play a role as an environmental trigger of PD [20, 21]. LPS signal through Toll-like receptors (TLR), a family of receptors involved in pathogen recognition and host defense. More precisely, LPS activate the nuclear factor kappa B (NF-κB) inflammatory pathway via TLR4 binding. A single systemic [13, 20] or intracerebral [22, 23] LPS injection replicates neuroinflammation features typically observed in PD, such as microglia activation and cytokine and iNOS production, associated to a chronic progression of dopaminergic neuron degeneration.

The Toll-interacting protein (Tollip) is a ubiquitously expressed protein, first identified in 2000 through two-hybrid screening using IL-1 receptor accessory protein as the bait [24]. The authors showed that Tollip forms a constitutive complex with the interleukin-1 receptor-associated kinase 1 (IRAK1). Upon interleukin 1 beta (IL-1β) stimulation, this complex is recruited, leading to the dissociation of Tollip from IRAK1 which transmits the IL-1β-induced signal. In addition, Tollip participates in the endolysosomal degradation of the IL-1R [25]. Accordingly, Tollip overexpression impaired IL-1β-induced activation of NF-κB [24], suggesting an inhibitory role in inflammatory signaling. Two subsequent in vitro studies showed that Tollip decreased Toll-like receptor 4 and 2 (TLR4 and TLR-2)-mediated inflammation by directly interacting with these receptors, supporting a pivotal role in the innate immune response [26, 27]. Accordingly, Tollip KO mice showed an increased susceptibility to dextran sulfate sodium-induced colitis [28].

Tollip immunomodulatory properties have been mainly investigated at the peripheral level and only limited studies have been conducted in the central nervous system. However, the brain has been described as the third region that displays the highest Tollip messenger RNA (mRNA) densities in human [29]. In addition, a tissue-based map of human proteome indicates that the cerebral cortex may display the highest density of Tollip protein, together with the epididymis and pancreas [30]. A limited number of observations suggest a potential role of Tollip in the brain. A microarray study reports downregulation of Tollip mRNA together with an upregulation of microglia- and perivascular macrophage-activating genes in brains of aging- and Alzheimer’s disease patients [31]. In addition, increased Tollip expression has been associated with a better outcome in a mouse model of stroke [32] whereas another group reported a deleterious effect of Tollip neuronal expression in a mouse model of cerebral ischemia/ reperfusion [33]. Finally, an in vitro study showed that the phosphatase and tensin homolog (PTEN)-induced putative kinase 1 (PINK1), well-known to be linked to an early-onset form of familial Parkinson’s disease (PD), binds Tollip [34]. The authors proposed that PINK1 stimulates IL-1β-induced signaling via suppression of Tollip inhibitory action. Despite these observations, a role of Tollip in the modulation of inflammation in the central nervous system has never been explored.

The *Tollip* gene contains six exons, three isoforms in the mouse (Tollip.a, .b, and .c) and four in humans (TOLLIP.A, .B, .C, and .D) resulting from alternative transcripts. These isoforms are highly conserved between the two species for some variants (94% between Tollip.a and TOLLIP.A and 92% between Tollip.c and TOLLIP.D) [35] (for review [36]). Furthermore, in the brain of C57BL/6J mice, the canonical Tollip.a mRNA is abundantly expressed and has been found in neurons, astrocytes, microglia, and endothelial cells at the seventh post-natal day [37], making the C57BL/6J mice suitable models to investigate the role of Tollip in the central nervous system.

The potential role of Tollip in the substantia nigra susceptibility to inflammation has never been evaluated. In the current study, we aimed at investigating (i) whether the Tollip protein is expressed in the substantia nigra and (ii) whether Tollip deletion (using Tollip knockout mice) may enhance LPS-mediated neuroinflammation.

## Methods

### Animals

All procedures and methods were approved by the Cantonal Veterinary Service for animal experimentation (SCAV-EXPANIM; authorization #VD2388.3). The animals had access to food and tap water *ad libitum* with a constant 12-h light/dark cycle. C57BL/6J Tollip knockout (KO) and wild-type (WT) mice were generously provided by Michel Maillard (Service of Gastroenterology and Hepatology, Department of Medicine, Lausanne University Hospital, Lausanne, Switzerland). After having observed that some of these mice carried a mutation on the alpha-synuclein gene, strongly linked to neurodegeneration and neuroinflammation in PD, we backcrossed mice onto the C57BL/6J RccHSD background strain in order to guarantee that all mice are alpha-synuclein +/+. Genetic backgrounds were checked by genotyping according to previously described protocols [38]. A total of 66 mice were used in this study according to the repartition described into the figure legends. Since our aim was to recapitulate pathogenic events related to PD, we have used middle-aged mice, i.e., at least 9 months old. Due to constraints in transgenic mice breeding, in order to work with age-matched groups for each experiment, we sometimes had to use animals of different sexes.

### Plasmids

The NF-κB-inducible self-complementary rAAV plasmid pSC-NF12d1-eGFP has been previously described [39]. Briefly, it contains a chimeric promoter consisting in a minimal CMV promoter flanked by 12 copies of the NF-κB responsive sequence. The pSC-NF12d1-eGFP-CMV-mCherry, a plasmid expressing enhanced green fluorescent protein (eGFP) under the control of the NF-κB-inducible promoter and constitutively expressing the mCherry, was constructed in order to allow controlling stereotactic injections.

### Viral vector

The NF-κB-inducible rAAV vector was produced by triple co-transfection of HEK-293T cells (thirty 10-cm plates seeded 24 h before transfection with 5 × 10^6^ cells) with (i) the pSC-NF12d1-eGFP-CMV-mCherry vector plasmid (4 μg/plate), (ii) a plasmid carrying the AAV serotype 2 Rep gene (Rep2) and a serotype 9 Cap gene harboring a mutation in a surface tyrosine (pXR9-2Y-F) which reduces viral particle proteosomal degradation (a kind gift from D. Dalkara [40]) (2 μg/plate), and (iii) an adenoviral helper plasmid (pAd-Helper; Stratagene) (5 μg/plate).

Fifty hours post transfection, the medium was discarded and the cells were harvested by low-speed centrifugation and resuspended in Tris pH 8.0, NaCl 0.1 M. After 3 cycles of freezing/thawing, the lysate was clarified by 30-min centrifugation at 10,000 *g*, treated with benzonase (50 units/mL, Sigma) at 37 °C for 30 min, and centrifuged at 10,000 *g* for 30 min to eliminate the residual debris. The virus was further purified by iodixanol gradient, buffer exchange, and microconcentration according to a previously described method [41] except the chromatography step which was omitted due to its poor recovery for AAV9. For clarity purposes, this viral vector has been designated hereafter as rAAV9-NRE-eGFP. The genomic titer of the recombinant virus was evaluated using real-time PCR as previously described [42]. The titer was 5 × 10^13^ viral genomes (vg)/mL.

### Stereotactic injections

Mice were anesthetized with ketamine 100 mg/kg (Ketasol, Graeub AG) and xylazine 10 mg/kg (Rompun®, Bayer) solutions and immobilized in the stereotactic frame. Animals received unilateral infusion of 1 μL of LPS at a concentration of 0.1 μg/μL (Sigma; *Escherichia coli* 026:B6) diluted in Dulbecco’s phosphate-buffered saline (DPBS) (Biowhittaker®, Lonza) solution. LPS from *E. coli* origin have a conically shaped lipid A portion and act as a specific ligand of the TLR4 [43]. Stereotaxic coordinates used were AP - 2.8 mm, ML - 1.3 mm, and DV - 4.5 mm, according to Bregma for LPS and AAV injections. The injection rate was 0.5 μL/min for LPS injection and 0.2 μL/min for rAAV injection. The needle (34 Gauge) was left in place for additional 5 min before removal. Control animals received 1 μL of DPBS. The animals were sacrificed 6 h after LPS injection.

### Real-time reverse transcription polymerase chain reaction (RT-qPCR)

#### Tissue collection

Six hours after unilateral LPS injection, mice were euthanized with an overdose of pentobarbital (30 mg/mL in 0.9% NaCl, Esconarkon®). The brain was removed and dissected on ice using stainless steel brain matrix (Stoelting Co, USA) and single-edge blades. The ventral part of the midbrain containing the substantia nigra pars compacta (SNc) was collected on three coronal sections at 1-mm intervals (Additional file 1: Figure S2b). The samples were immediately frozen in liquid nitrogen and stored at −80 °C.

#### RNA extraction and cDNA preparation

Total RNA was extracted from tissue samples using RNeasy® Kit (Qiagen) following manufacturer’s instructions. Quantity and purity of samples were assessed by absorbance measurement with a NanoDrop spectrophotometer (Thermo Fisher Scientific, USA).

The reverse transcription reaction was carried out using the High-Capacity cDNA Reverse Transcriptase kit as already described [44], using reagents and protocols from Thermo Fisher. Thermocycling conditions were the following: 25 °C for 10 min, 37 °C for 120 min, 85 °C for 5 min, and a final step at 4 °C ad infinitum.

#### Quantitative PCR

Semi-quantitative real-time PCR were carried out by ABI PRISM 7700 Detection system (ABI, Foster City, CA, USA) using the SYBR® Select Master Mix (Life Technologies, USA) according to the manufacturer’s protocols. For mRNA quantification of eGFP (green fluorescent protein) 4 ng of total complementary DNA (cDNA) was loaded per reaction, and 2.4 ng/reaction was used for detection of IL-1β and inducible nitric oxide synthase (iNOS). For mRNA quantification of glyceraldehyde 3-phosphate dehydrogenase (GAPDH), β-actin, glial fibrillary acidic protein (GFAP), and neurofilament H (NFH), 0.4-ng total cDNA per reaction was used. Real-time PCR was performed in a total volume of 10 μL of SYBR® Green Master Mix (ABI, Foster City, CA, USA) containing 150 μM of forward and reverse primers for eGFP and 300 μM for all other genes. Thermocycling conditions were 50 °C for 2 min, 95 °C for 10 min, and 40 cycles of 15 s 95 °C and 1 min of 60 °C, following by a dissociation stage. Gene specific primers used are listed in Table 1. The standard curve model was used to calculate the mRNA expression relative to a serial dilution of a pool comprising all samples. Both β-actin and GAPDH genes were used as internal controls for each sample and all reactions were run in technical triplicates. Post-PCR melting curves were analyzed to ensure primer specificity. For each sample, mRNA values were normalized by the value given by the housekeeping gene (β-actin + GAPDH)/2 that was similar between all groups.

**Table 1:** Mouse primers used for real-time qPCR analysis

Gene	Sequences of primers
β-Actin	Forward 5′-GCCCTGAGGCTCTTTTCCAG-3′
	Reverse 5′-TGCCACAGGATTCCATACCC-3′
CD68	Forward 5′-TTCTGCTGTGGAAATGCAAG-3′
	Reverse 5′-AGAGGGGCTGGTAGGTTGAT-3′
GAPDH	Forward 5′-TGTGGATGGCCCCTCTGGAA-3′
	Reverse 5′-TCAGATGCCTGCTTCACCAC-3′
GFAP	Forward 5′-CCAGCTTACGGCCAACAGT-3′
	Reverse 5′-TGGTTTCATCTTGGACTTCTG-3′
IFN-γ	Forward 5′-TGGCTCTGAAGGATTTTCATC-3′
	Reverse 5′-TCAACTGGCATAGATGTGGAAGAA-3′
IL-1β	Forward 5′-TCGAGGCCTAATAGGCTCATCT-3′
	Reverse 5′-GCTGCTTCAGACACTTGCACAA-3′
IL-6	Forward 5′-TGTTCTCTGGGAAATCGTGGAA-3′
	Reverse 5′-GCAAGTGCATCATCGTTGTTCA-3′
IL-10	Forward 5′-ACCTGCTCCACTGCCTTGCT-3′
	Reverse 5′-GGTTGCCAAGCCTTATCGGA-3′
iNOS	Forward 5′-CATTGGAAGTGAAGCGTTTCG-3′
	Reverse 5′-CAGCTGGGCTGTACAAACCTT-3′
NFH	Forward 5′-CACCAAGGAGTCACTGGAG-3′
	Reverse 5′-TGCTGAATAGCGTCCTGGTA-3
TNF-α	Forward 5′-CCTCTTCTCATTCCTGCTTGTGG-3′
	Reverse 5′-GGCCATTTGGGAACTTCTCATC-3′
eGFP	Forward 5′-CAAAGACCCCAACGAGAAGC-3′
	Reverse 5′-CTTGTACAGCTCGTCCATGC-3′
Arg-1	Forward 5′-GAACACGGCAGTGGCTTTAAC-3′
	Reverse 5′-TGCTTAGCTCTGTCTGCTTTGC-3′
CD32	Forward 5′-AATCCTGCCGTTCCTACTGATC-3′
	Reverse 5′-GTGTCACCGTGTCTTCCTTGAG-3′
CD206	Forward 5′-TCTTTGCTTTCCAGTCTCC-3′
	Reverse 5′-TGACACCCAGCGGAATTTC-3′

### Immunohistochemistry

Mice were perfused transcardially with PBS pH 7.4 (Bichsel AG) followed by ice-cold 4% paraformaldehyde in PBS pH 7.4 (PF4). Brains were post-fixed in PF4 overnight at 4 °C, cryoprotected during two successive days in 20 and 30% sucrose, and gradually frozen in isopentane/dry ice. Coronal sections (30 μm thick) were cut using a cryostat (Leica CM1850) and stored in anti-freeze medium (composed by glycerol, ethylene glycol, PBS, and water) at −20 °C.

Sections were washed in 10-mM Tris-buffered saline pH 7.6 (TBS). Non-specific binding was blocked using 5% bovine serum albumin (BSA, Sigma-Aldrich, USA) in TBS containing 0.5% of Triton-X100 (Sigma-Aldrich, USA) (THST). Sections were incubated overnight at 4 °C with the primary antibody in 1% BSA/THST followed by an incubation with secondary antibodies in THST for 1 h at room temperature. Slices were then mounted on microscope slides with Vectashield (Vector Laboratories, Burlingame, CA, USA) and stored at 4 °C in the dark. The labeling analyses were performed on three sections at the level of the SNc for each individual.

The co-immunostaining of the endogenous Tollip protein and the tyrosine hydroxylase enzyme (TH) was performed using a rabbit polyclonal antibody against the full-length Tollip protein (1:50; ab187198, Abcam) followed by a secondary donkey anti-rabbit antibody (1:1000; A2106, Molecular Probes) and a mouse anti-TH (1:1000; MAB318, Millipore) followed by a goat anti-mouse antibody (1:1000; A-11005, Molecular Probes). Oxidative and nitrosative stresses were assessed using a mouse monoclonal anti-8-oxo-dG (1:350; AMS Biotechnology, Bioggio-Lugano) and a mouse monoclonal anti-nitrotyrosine (1:1000; Chemicon International) antibody, respectively. Microgliosis was evaluated using a rat monoclonal anti-CD68 (1:1500; Abcam) and a goat polyclonal anti-Iba1 (1:1000; Abcam) antibody. Finally, iNOS was measured with a rabbit polyclonal anti-iNOS (1:500; Novus Biological) and NeuN with a mouse monoclonal anti-NeuN (1:2500; Millipore).

### Image analysis

Images were acquired with a Zeiss Confocal Microscope equipped with 310, 320, 340, and 363 Plan-NEOFLUAR objectives. All parameters were controlled with LSM510 software. For quantification of 8-oxo-dG, iNOS, and 3-NT immunostainings, images were acquired with a ×40 objective, at a resolution of 1024 × 1024 pixels. In each animal, quantification has been made on two fields per section, on three 30-μm-thick sections at the level of SNc. Analysis has been made with IMARIS 7.3 software (Bitplane AG, Switzerland). Results are expressed as number of positive cells/section or as a mean of fluorescence intensity/section. For Tollip immunostaining, quantification has been performed using a ×20 magnification. Analysis was performed using ImageJ v1.4 (National Institute of Health, USA).

### IL-1β ELISA

All mice underwent unilateral injection of 0.1-μg LPS according to the protocol described in the “Stereotactic injections” section. Six hours later, midbrain extracts from ipsilateral and contralateral side relative to the injection have been homogenized in Tper solution (Thermo Scientific), containing phosphatase and protease inhibitor cocktail (Roche). IL-1β enzyme-linked immunosorbent assay (ELISA) has been performed on 60 μg of total protein, using the kit Bioscience cat # 88-7013 according to the manufacturer’s instructions.

### Statistical analysis

Data analyses and graphs were performed using GraphPad Prism 6 software for Windows (GraphPad Software Inc., San Diego, CA, USA). For mRNA expression, ELISA, and immunohistochemistry analysis, comparisons were performed by two-way ANOVA followed by Tukey’s post hoc test.

Results were expressed in the text as mean ± SEM, and statistical significance was established for a *P* value ≤0.05.

## Results

### Tollip protein is highly expressed in dopaminergic neurons

**Fig. 1:**
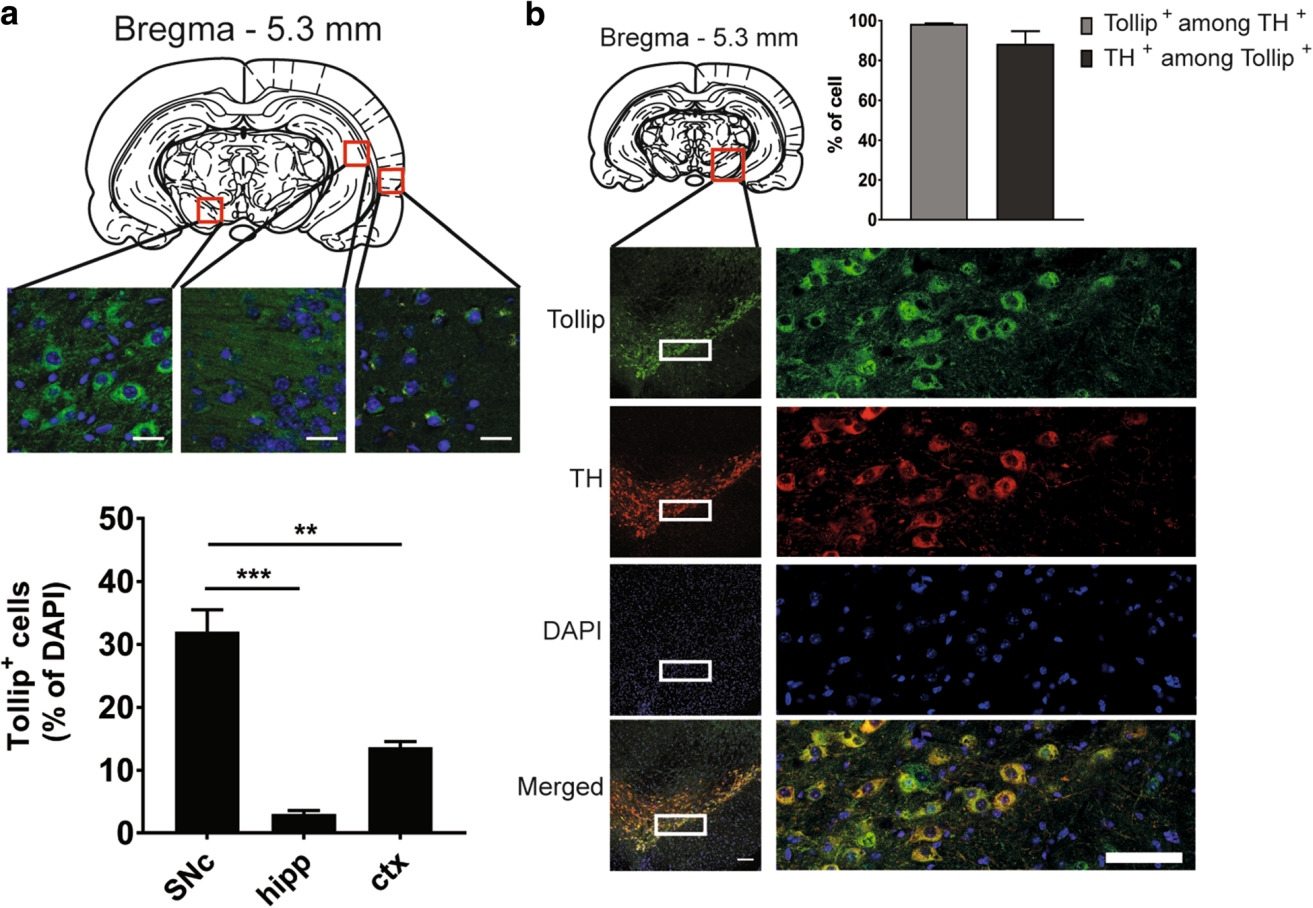
Tollip is highly expressed in dopaminergic neurons. **a** Representative Tollip (in *green*) and DAPI staining (in *blue*) in 10-month-old female Tollip WT mice. *Up* - position of the analyzed sections in the Paxinos Atlas [84]. *Inset* shows the substantia nigra pars compacta (SNc), the hippocampus (hipp), and the cortex (ctx) analyzed below. Magnification = ×63. Scale bar = 10 μm. *Bar graphs* below represent the number of Tollip-positive cells relative to DAPI-positive cells. (*n* = 3 WT mice); **P < 0.01; ***P < 0.001; one-way ANOVA, Holm–Sidak’s post hoc test. **b** Representative Tollip (in *green*) and tyrosine hydroxylase (TH) (in *red*) immunostainings and DAPI staining (in *blue*) in the SNc of 9-month-old male Tollip WT mice. Magnification = ×20. Scale bar = 50 μm. *White insets* show the region photographed on the right, at high magnification (×63); scale bar = 50 μm. *Bar graph* indicates that 98.3 ± 0.3% of TH-positive cells display also Tollip immunostaining and 88.3 ± 6.3% of Tollip-positive cells are TH immunopositive

Unexpectedly, the percentage of Tollip-immunopositive cells relative to the DAPI-positive cells in the substantia nigra pars compacta (SNc) (32.0 ± 3.5%) was 10- and 2-fold higher than those found respectively in the hippocampus (3.0 ± 0.6%) and the cortex (13.7 ± 0.9%) (Fig. 1a). Tollip immunostaining displayed the typical shape of the SNc/ventral tegmental (VTA) area (Fig. 1b). It should be noted that this was similarly observed both in male and female middle-aged mice. The co-staining with anti-TH antibody showed in addition that Tollip protein is particularly abundant in dopaminergic neurons, as 98.3 ± 0.3% of TH-positive cells also express Tollip (Fig. 1b). This novel observation points to a potential role of Tollip in dopaminergic neurons. However, Tollip is also detected albeit at a lower level, outside the SNc in cells not labeled by anti-TH antibodies (Additional file 2: Figure S1a, b; see arrows). The identity of these cells remains to be determined. As expected, no Tollip staining was observed in Tollip KO mice (Additional file 2: Figure S1c).

Since an increase of Tollip protein detection after LPS stimulation of immune cells in vitro has been previously described [45], we tested whether Tollip expression could be increased after LPS injection in the midbrain of Tollip WT mice. Brains were analyzed at 6 h post - LPS injection. The pattern of Tollip protein expression was not modified by LPS (0.1 μg) treatment as compared with the non-injected side (Additional file 1: Figure S2a). Furthermore, quantification of the Tollip mRNA by RT-qPCR after injection of increasing LPS doses confirmed that Tollip gene expression in the midbrain was not stimulated by LPS (Additional file 1: Figure S2b).

### Effect of LPS (0.1 μg) on cytokines mRNA expression in the midbrain of Tollip-deficient mice

Our goal was to evaluate the effect of Tollip deletion on the early neuroinflammatory phase induced by an intra-nigral LPS injection.

First, increasing LPS doses were unilaterally injected in the midbrain of adult Tollip WT mice and the expression of pro-inflammatory cytokines evaluated 6 h later by RT-qPCR. As shown in Additional file 3: Figure S3, a LPS dose-dependent increase of IL-1β, interleukin 6 (IL-6), and tumor necrosis factor alpha (TNF-α) mRNA expression was observed.

Since our goal was to investigate whether Tollip deficiency could exacerbate the effect of the LPS injection, we wanted to avoid using a saturating LPS dose which could have possibly masked the effect of Tollip deletion. Therefore for further analyses, a submaximal LPS dose of 0.1 μg was chosen.

**Fig. 2:**
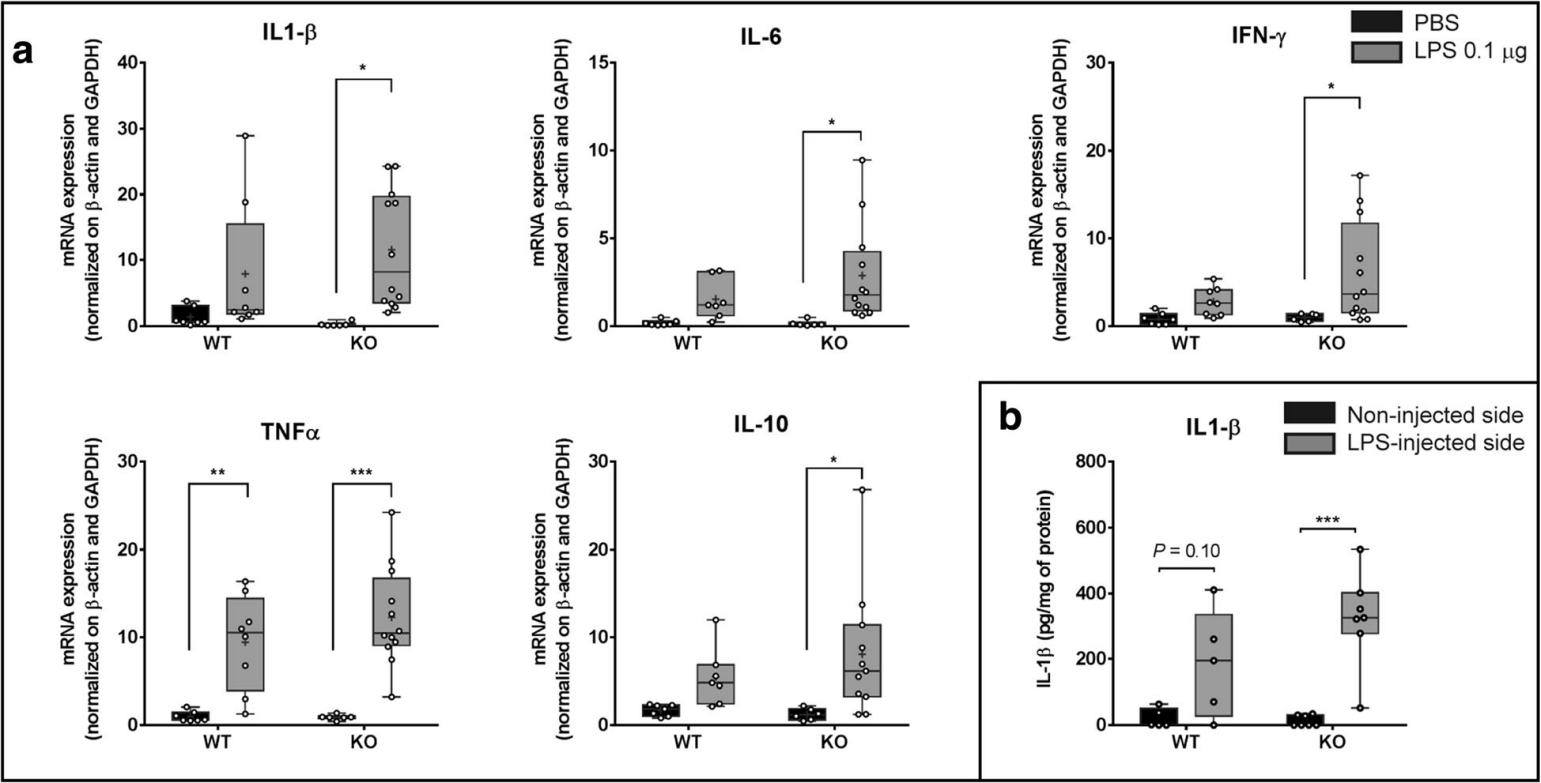
Inflammatory mediators are increased in the midbrain of Tollip-deficient mice 6 h after LPS intra-nigral injection. **a** Eleven-month-old female mice underwent PBS (1 μL) or LPS (0.1 μg in 1 μL) injection (*n* = 7 and *n* = 8 respectively for WT mice and *n* = 6 and *n* = 12 respectively for the Tollip KO mice). mRNA amounts in midbrain extracts are normalized by β-actin and GAPDH. **b** IL-1β protein quantification by ELISA on 60 μg of midbrain protein extracts from the injected (LPS 0.1 μg) and non-injected sides. *n* = 5 for WT and 7 for KO 11-month-old mice. *Box-and-whisker graphs* show median, min., and max. values and 25th and 75th percentiles. Plots represent individual values and means are represented as a “+.” *P < 0.05; **P < 0.01; ***P < 0.001 versus PBS for **a**. and versus non-injected side for **b**. Two-way ANOVA followed by a Tukey’s post hoc test

The basal and LPS-induced patterns of cytokine expression in the brain of aged Tollip WT and Tollip KO mice were then compared. In the absence of inflammatory challenge, no difference was observed between Tollip WT and KO mice, suggesting that the absence of Tollip does not induce a spontaneous inflammation in the brain (Fig. 2, black bars). As expected, LPS intra-nigral injection increased the level of transcription of all cytokines tested (Fig. 2, gray bars). However, a differential increase in Tollip WT versus Tollip KO mice was observed for most of the cytokines. Indeed, in Tollip WT mice, although the mRNA expression of IL-1β, IL-6, IFN-γ, TNF-α, and IL-10 was increased respectively 6-, 9-, 3-, 10-, and 3-fold in response to LPS, this effect did not reach significance, except for TNF-α (Fig. 2a). In contrast, in Tollip KO mice, LPS elicited a significant increase of all cytokines: 40-, 19-, 6-, 14-, and 6-fold respectively as compared with PBS-injected mice (*P* < 0.05 for IL-1β, IL-6, IFN-γ, IL-10, and *P* < 0.001 for TNF-α). However, the difference between LPS-injected side in Tollip KO and Tollip WT mice did not reach significance. IL-18 was not altered in any condition (data not shown).

In order to evaluate whether differences in transcript levels were also detectable at the protein level, we investigated whether Tollip deletion had any impact on IL-1β abundance upon intra-nigral LPS injection (Fig. 2b). IL-1β concentration in midbrain extracts from injected and non-injected sides has been evaluated by ELISA. Consistently with our prior data, whereas the LPS-induced IL-1β increase did not reach statistical significance in WT mice (*P* = 0.1), it was significant in Tollip KO mice (*P* < 0.001). However, direct comparison of protein abundance between Tollip WT and KO mice failed to reach significance (184 ± 74 pg/mg (Tollip WT) versus 324 ± 55 pg/mg (Tollip KO), *P* = 0.17, Fig. 2b).

NFH (neurofilament) mRNA was not altered in any condition, suggesting that the early-phase inflammatory response did not result in neuronal toxicity (data not shown).

In conclusion, genetic deletion of Tollip did not affect baseline inflammatory cytokine pattern, but led to increased cytokine mRNA expression upon low-dose LPS injection in the substantia nigra.

### Tollip KO mice display a higher NF-κB activation in an inflammatory context

**Fig. 3:**
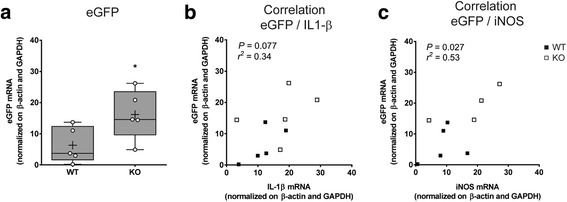
Tollip KO mice display a higher NF-κB-activation. Thirteen-month-old male mice underwent intra-nigral injection of rAAV9-NRE-eGFP (5 × 10^8^ viral genomes) followed by LPS (0.1 μg) injection 2 weeks later. Analyses were performed at 6 h post-injection of LPS (*n* = 5 mice per group). eGFP mRNA amounts in midbrain extracts were normalized by β-actin and GAPDH. **a** Results are represented as *box* and *whiskers* that show median, min., and max. values, 25th and 75th percentiles. Plots represent individual values and means are represented as a “+.” *P < 0.05 versus WT mice using a Mann–Whitney test. **b**–**c** *Graph* shows correlation between eGFP mRNA and IL-1β mRNA (**b**) and iNOS mRNA (**c**) in the injected side of Tollip KO (*empty square*) and WT (*filled square*) mice. The *r* squared was obtained using Pearson’s correlation

The observed altered cytokine response upon LPS challenge in Tollip KO mice suggests that Tollip is involved in the regulation of NF-κB activity upon LPS challenge in the brain. In order to investigate whether a higher NF-κB activation is observed in the absence of Tollip, a NF-κB-inducible rAAV9-NRE-eGFP viral vector was injected in the SNc of Tollip WT (*n* = 5) and Tollip KO mice (*n* = 5), 2 weeks before LPS challenge (Fig. 3). Six hours after infusion of LPS (0.1 μg), a higher amount of eGFP mRNA in the midbrain of Tollip KO mice was measured, as compared to WT mice (2.5-fold, *P* < 0.05) (Fig. 3a). eGFP mRNA amount tended to correlate with IL-1β mRNA (*P* = 0.08, Fig. 3b) and correlated with iNOS mRNA (*P* < 0.05, Fig. 3c). This result shows that Tollip KO mice display a higher NF-κB activation than Tollip WT mice.

### LPS-induced iNOS expression is significantly increased in the midbrain of Tollip-deficient mice

**Fig. 4:**
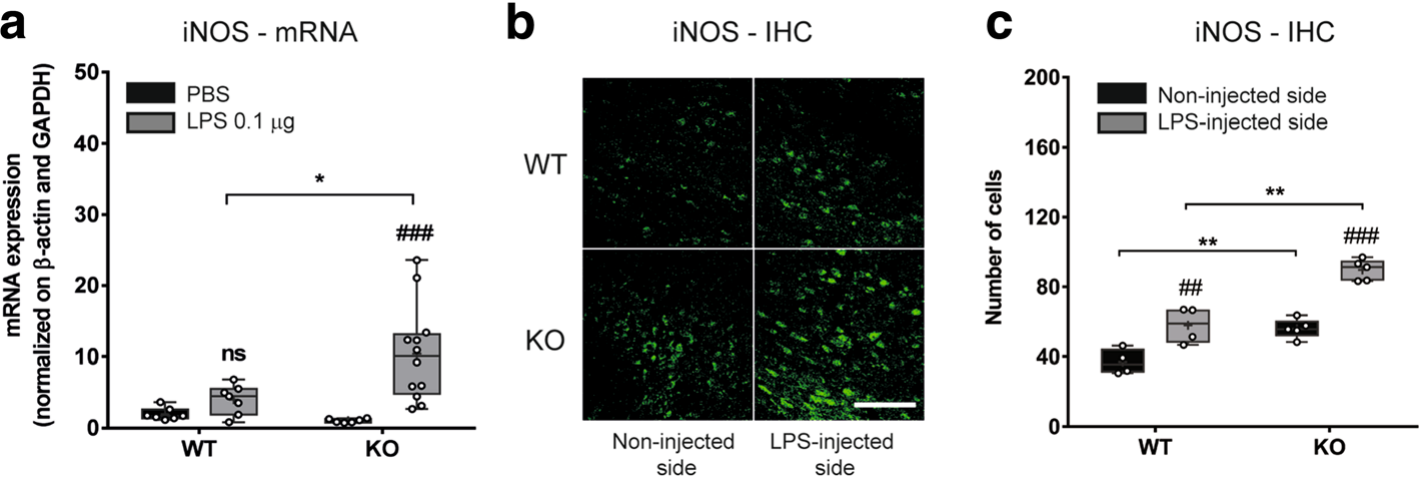
iNOS is increased in the midbrain of Tollip-deficient mice 6 h after LPS intra-nigral injection. **a** Eleven-month-old female mice underwent PBS (1 μL) or LPS (0.1 μg in 1 μL PBS) injection (*n* = 7 and *n* = 8 respectively for WT mice and *n* = 6 and *n* = 12 respectively for Tollip KO mice). mRNA amounts in midbrain extracts were normalized by β-actin and GAPDH. *P < 0.05 versus WT mice and ^###^ *P* < 0.001 versus PBS. **b** Representative iNOS immunostaining in contralateral and ipsilateral side of the LPS (0.1 μg) injection, in WT and Tollip KO 15-month-old male mice. Magnification = ×40. Scale bar = 30 μm. **c** Quantification of iNOS-positive cells, performed on two fields in the injected area, on three sections per animal (*n* = 4 mice in WT and 5 mice in KO group). **P < 0.01 versus WT mice and ^##^ *P* < 0.01 and ^###^ *P* < 0.001 versus non-injected side. *Box* and *whiskers* show median, min., and max. values, 25th and 75th percentiles. Plots represent individual values and means are represented as a “+.” Two-way ANOVA followed by a Tukey’s post hoc test

We next wanted to address whether the observed increased NF-κB activity was associated with induction of inducible nitric oxide synthase (iNOS), another NF-κB target gene [46] that is related to PD neuropathology [16] (Fig. 4). As for cytokine mRNA measurements, submaximal LPS doses (0.1 μg) were not sufficient to significantly increase iNOS mRNA in Tollip WT mice, whereas it was in Tollip KO mice (by 10-fold as compared with PBS-injected group, *P* < 0.001, Fig. 4a). In Tollip KO mice, this increase was 2.6-fold larger than in WT mice (*P* < 0.05).

In order to confirm this observation, we assessed iNOS protein expression by immunohistochemistry of brain tissue from mice subjected to LPS (0.1 μg) stereotactic injection (Fig. 4b). In both Tollip WT and KO mice, the number of iNOS-positive cells was higher in the ipsilateral (LPS-injected) side than in the contralateral side (*P* < 0.01 for WT mice and *P* < 0.001 for KO mice, Fig. 4c). This effect was specific to LPS and not due to the surgery as demonstrated by the absence of iNOS immunostaining in PBS-injected midbrains (Additional file 4: Figure S4). The number of iNOS-positive cells was 58 ± 11 in LPS-injected side in WT mice versus 90 ± 6 in Tollip KO mice, confirming that Tollip KO mice displayed an increased iNOS expression level as compared to WT mice (*P* < 0.01). Surprisingly, the quantification in the side contralateral to the LPS injection revealed also a higher number of iNOS-positive cells in KO mice (*n* = 56 ± 2) than in WT mice ((*n* = 37 ± 4), *P* < 0.05). This effect could possibly be attributed to an overall higher reactivity of the whole brain in response to the unilateral LPS injection in Tollip KO mice.

### Effect of Tollip deletion on protein nitrosylation

**Fig. 5:**
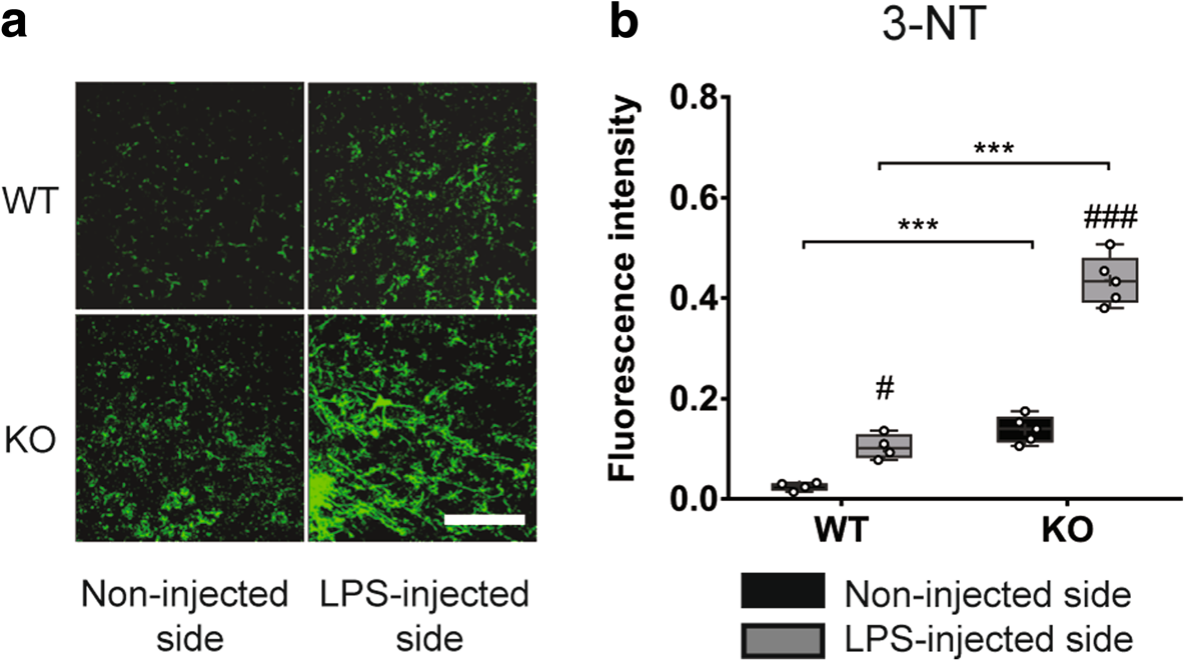
Nitrotyrosine immunostaining is increased in the midbrain of Tollip-deficient mice 6 h after LPS intra-nigral injection. **a**. Representative nitrotyrosine immunostaining in non-injected and LPS (0.1 μg) - injected side in WT and Tollip KO 15-month-old male mice. Magnification = ×40. Scale bar = 30 μm. **b** Quantification of fluorescence intensity performed on two fields at the injected area, on three sections per animal (*n* = 4 in WT and 5 mice in KO group). *Box* and *whiskers* show median, min., and max. values, 25th and 75th percentiles. Plots represent individual values and means are represented as a “+.” ^#^ *P* < 0.05; ^###^ *P* < 0.001 versus non-injected side and ^***^ *P* < 0.001 versus WT mice. Two-way ANOVA followed by a Tukey’s post hoc test

iNOS increase has been demonstrated to lead to a high level of nitric oxide (NO) production [47]. In the presence of oxidative stress, NO may produce peroxynitrite radicals, a powerful oxidant that is responsible for nitrosylation of proteins. In order to further evaluate the impact of iNOS increase a quantification of nitrotyrosine, a commonly accepted marker of nitrosative stress found to be increased in PD patients CSF and serum [48], was performed (Fig. 5a). As expected, LPS induced an increase of nitrotyrosine immunostaining in Tollip WT (*P* < 0.05) and in Tollip KO mice (*P* < 0.001), as compared with the contralateral side. In the LPS-injected side, fluorescence intensity of the nitrotyrosine immunostaining was 4-fold increased in Tollip KO mice versus Tollip WT mice (*P* < 0.001, Fig. 5b). As for iNOS quantification, nitrotyrosine staining in the side contralateral to the LPS injection was higher in Tollip KO than in Tollip WT mice (*P* < 0.001). These data suggest that Tollip KO mice are highly predisposed to peroxynitrite formation.

### Effect of Tollip deletion on oxidative stress

**Fig. 6:**
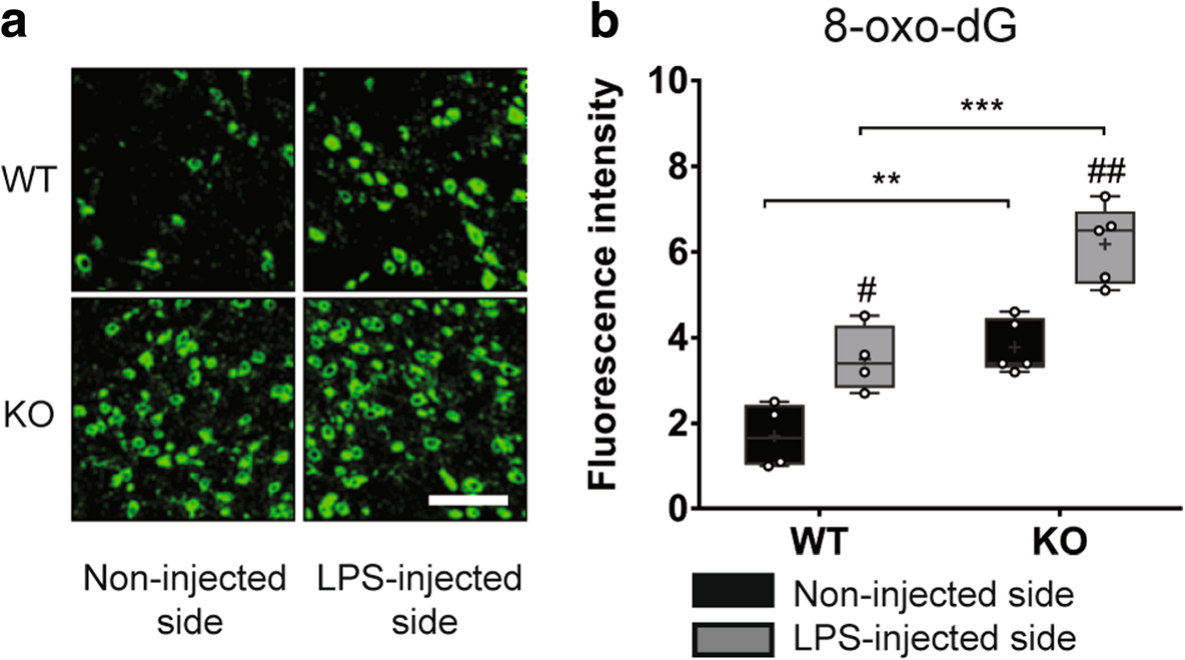
8-oxo-2′-deoxyguanosine (8-oxo-dG) immunostaining is increased in midbrain of Tollip-deficient mice 6 h after LPS intra-nigral injection. **a** Representative 8-oxo-DG immunostaining in the non-injected and LPS (0.1 μg)-injected side in WT and Tollip KO 15-month-old male mice. Magnification = ×40. Scale bar = 30 μm. **b** Quantification of fluorescence intensity performed on two fields in the injected area, on three sections per animal (*n* = 4 in WT and 5 mice in KO group). *Box* and *whiskers* show median, min., and max. values, 25th and 75th percentiles. Plots represent individual values and means are represented as a “+.” ^#^ *P* < 0.05; ^##^ *P* < 0.01 versus non-injected side and ^**^ *P* < 0.01; ^***^ *P* < 0.001 versus WT mice. Two-way ANOVA followed by a Tukey’s post hoc test

In order to validate the hypothesis of a high level of oxidative stress in Tollip KO mice, 8-oxo-2′-deoxyguanosine (8-oxo-dG), a common marker of oxidized DNA, has been measured in the midbrain (Fig. 6a). Six hours after LPS injection, 8-oxo-dG immunoreactivity was mainly observed in the cytoplasm, suggesting 8-oxo-dG accumulation in mitochondrial DNA in WT mice (*P* < 0.05) as well as in KO mice (*P* < 0.01). Quantification showed that fluorescence intensity was significantly increased in KO mice, on both sides, ipsilateral and contralateral to the LPS injection (*P* < 0.001 and *P* < 0.01 respectively, Fig. 6b). This result suggests that Tollip KO mice are more susceptible to oxidative stress, as revealed by increased DNA oxidation in response to a pro-inflammatory challenge than WT mice.

### Effect of Tollip deletion on early-phase glial activation

**Fig. 7:**
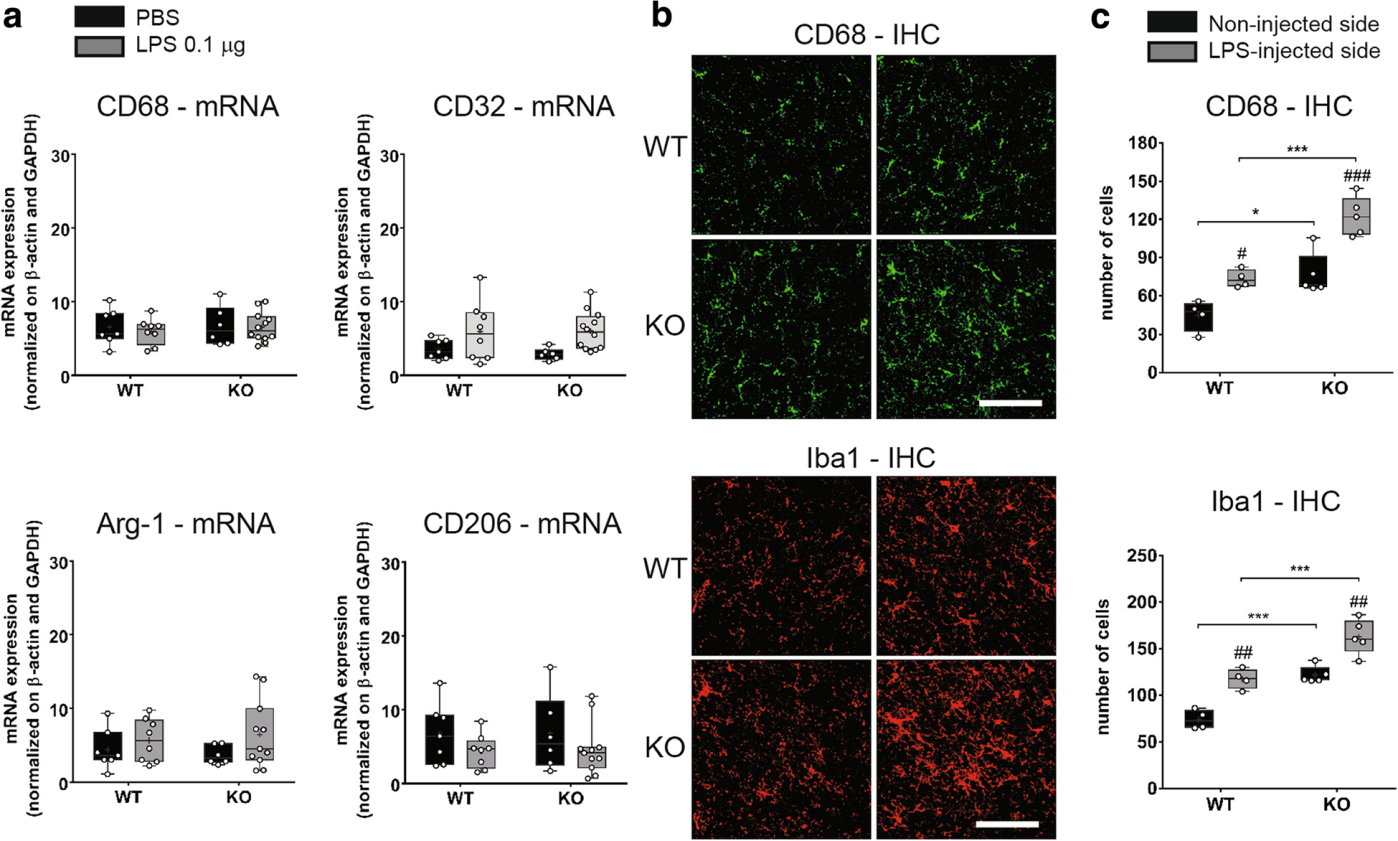
Acute microglial response to LPS. **a** Eleven-month-old female mice underwent PBS (1 μL) or LPS (0.1 μg) injection (*n* = 7 and *n* = 8 respectively for WT mice and *n* = 6 and *n* = 12 respectively for the Tollip KO mice). mRNA amounts in midbrain extracts were normalized by β-actin and GAPDH. **b** Representative CD68 and Iba1 immunostainings in non-injected (*left*) and LPS (0.1 μg)-injected side (*right*) in WT and Tollip KO 15-month-old male mice. Magnification = ×40. Scale bar = 30 μm. **c** Quantification of CD68 and Iba1-positive cells, performed on two fields in the injected area, on three sections per animal (*n* = 4 in WT and 5 mice in KO group). Results are represented as *box* and *whiskers* that show median, min., and max. values, 25th and 75th percentiles. Plots represent individual values and means are represented as a “+.” ^#^ *P* < 0.05; ^##^ *P* < 0.01; ^###^ *P* < 0.001 versus non-injected side and ^*^ *P* < 0.05; ^***^ *P* < 0.001 versus WT mice. Two-way ANOVA followed by a Tukey’s post hoc test

As TLR4 and iNOS are mainly expressed in astrocytes and macrophages [49–52], we hypothesized that excessive glial activation may account for the high oxidative and nitrosative stress in Tollip-deficient mice. A series of microglial markers have been measured at the transcriptional level using qPCR analysis (Fig. 7a) and at the protein level using immunohistochemistry (Fig. 7b, c). Moreover, as it is widely accepted that activated microglia exert dual functions, i.e., pro-inflammatory (M1) and anti-inflammatory (M2); we also evaluated whether Tollip deletion may induce a shift toward one of these two phenotypes. We measured Iba1 which is a widely used marker of microglial activation, CD32 and CD68 which are associated with M1 phenotype, and Arg1 and CD206 which are considered as M2 phenotype markers [53].

LPS is known to elicit an “M1-type” response [54]. However, none of the M1-associated mRNA markers were significantly altered 6 h after LPS injection (Fig. 7a). LPS (0.1 μg) being a submaximal dose, we have then used higher concentrations of LPS, but no changes of CD68 mRNA have been detected at any LPS dose (Additional file 5: Figure S5). In contrast to those expression studies, immunostaining showed an increase in CD68 protein abundance following LPS stimulation in both Tollip WT and KO mice, with a 67% increase of the number of CD68-positive cells in the LPS-injected side in KO mice as compared with WT mice (*P* < 0.001, Fig. 7b, c). Iba1 immunostaining was also increased in the injected side in Tollip WT and KO mice (*P* < 0.01), with a 39% increase of Iba1-positive cells in the LPS-injected side in KO mice as compared with WT mice (Fig. 7b, c). As for iNOS, nitrotyrosine, and 8-oxo-dG markers, CD68 and Iba1 stainings in the side contralateral to the LPS injection were higher in Tollip KO than in Tollip WT mice (*P* < 0.05 and *P* < 0.001, respectively).

No astroglial activation, as measured by quantifying GFAP mRNA, was observed in both genotypes (data not shown).

In conclusion, the mRNA expression of microglia markers was not significantly altered at 6-h post-injection of LPS (0.1 μg) in either of the genotypes, and no polarization of macrophages/microglia toward M1 or M2 subtypes was observed. Nevertheless, the CD68 and Iba1 immunostainings indicated higher microglia activation in Tollip KO mice as compared with Tollip WT mice, suggesting a higher reactivity of Tollip KO mice to LPS challenge.

## Discussion

We report in this study a distinctive high density of Tollip protein in dopaminergic neurons of the SNc and VTA. This novel observation suggests that in spite of being ubiquitous in the brain, Tollip may play a major role in dopaminergic cells. This result was unpredictable from previous studies on human brain in which Tollip mRNA was detected in SNc but not in a larger amount relative to other structures [55, 56]. Thus, exploring the impact of Tollip in dopaminergic neurons appears of primary importance. Therefore, the effect of the absence of the Tollip gene on the inflammatory response to a LPS challenge in the mice SNc was studied.

In the current study, we used LPS at a one order of magnitude lower dose than previously described in models of PD [22, 23, 57]. Indeed, in chronic diseases, a very low-grade endotoxemia can lead to systemic inflammation [58]. Accordingly, in the brain, an acute low-dose LPS challenge was shown to induce a long-term sustained inflammatory state which can potentiate alpha-synuclein dysfunction in an animal model of PD [13]. Consequently, a low LPS dose is more likely to recapitulate a clinically relevant situation in which a mild inflammatory stimulus becomes deleterious in a context of increased susceptibility.

Our results show that Tollip controls LPS-mediated inducible NO synthase (iNOS) quantities. This was associated with a global tendency of increased cytokine induction in the absence of Tollip. This inflammatory response is in accordance with a TLR4-mediated acute inflammation in the brain [59]. Tollip has already been described as a negative regulator of TLR4 signaling [26, 27], modulating the early MyD88-dependent response in vitro (for review [60, 61]) and leading to decreased NF-κB activation. These observations support the idea that, as already observed in vitro in immune cells, Tollip regulates the NF-κB signaling pathway in the brain. Accordingly, we showed, using an NF-κB-inducible viral vector, that activation of NF-κB was higher in Tollip KO mice, presumably accounting for this higher inflammatory response. Our results suggest that insufficient Tollip expression may lead to an increased vulnerability to an inflammatory stimulus. Interestingly, Cribbs and colleagues reported a decrease in Tollip mRNA expression in the brain of aging humans and patients diagnosed with dementia [31], suggesting that the inflammatory response may be altered in the brain of these patients.

In our study, the inflammatory response to LPS likely involved resident cells, since recruitment of peripheral immune cells has been described to occur at later time points [62, 63]. This hypothesis is consistent with the fact that resident glial cells have been reported to express TLR4, the target of LPS, and iNOS [49, 51, 64]. iNOS activation as well as IL-1β, TNF-α, and IL-6 increases is typically associated with activated microglia with a M1-like phenotype. On the basis of mRNA analysis, no obvious increase of glial activation markers without any evidence for a shift toward M1 phenotype have been observed 6-h post-LPS injection in both genotypes. Contrarily to mRNA expression, CD68 and Iba1 protein analyses showed a stronger immunostaining in the SNc upon LPS challenge as compared with the non-injected side, in both genotypes. This discrepancy may be due to differences in sample composition between qPCR and immunohistological measurements. Indeed, whereas midbrain extracts for qPCR were prepared from large punches including but not limited to the SNc, the quantification of immunostainings was restricted to the SNc, thus allowing for more precision. Alternatively, sex difference may explain this discrepancy. In our studies, females and males have been respectively used for mRNA and immunohistochemistry experiments. Indeed, in a LPS-induced neurodegeneration model, a higher sensitivity of males versus females has been observed [65].

Moreover, the 6-h time point may be too early to detect a robust microglial markers increase. Indeed, previous studies in rats showed increased CD68 and CD11b/c [62] and CD11b and Iba1 immunostainings [63] at 12 or 15 h respectively but not at 5 h after intra-cerebral injection of LPS. Similarly, cortical GFAP markers were upregulated upon LPS injection at 24 h [50]. Thus, it remains to be determined if in our study, a more robust microglial and astrocytic activation could be detected at later time points. In the present study, the fact that microglia mRNA markers are not increased in Tollip KO mice as compared to WT mice upon LPS challenge may indicate that microglial cells participate but are not the only contributor to increased inflammation susceptibility. Additional mechanisms must be investigated.

LPS (0.1 μg) induced a larger increase in iNOS mRNA and protein amounts in Tollip KO mice as compared with WT mice. The iNOS enzyme generates high level of NO that plays an important role as a host defense mechanism [52]. However, in the presence of oxidative stress, it interacts with the superoxide anion radicals (O_2_^−^) to generate the free radical peroxynitrite (OONO), the main nitrating agent in vivo [66–68]. Interestingly, in our experimental setting, the iNOS increase correlated with an increase in nitrotyrosine immunostaining, a widely used marker of peroxynitrite formation. The deleterious effect of the cerebral nitric oxide release has been extensively reviewed [69–71]. Notably, nitration affects protein functions [72, 73] and a role of NO overproduction in PD patients was already suggested 30 years ago [16, 17, 74]. Therefore, the greater propensity of Tollip KO mice to generate nitrosative stress indicates that Tollip may constitute a potential target for neuroprotective therapies aimed at reducing iNOS-mediated damage.

Tollip is also able to interact with a wide range of proteins that are not directly implicated in the regulation of TLR-mediated NF-κB pathway, and the fact that Tollip deletion might elicit effects that are not directly related to NF-κB activation cannot be ruled out. A recent study revealed that Tollip promoted neuronal apoptosis following ischemia/reperfusion injury in rodents, by directly inhibiting Akt signaling in a TLR-independent manner [33]. In addition, Tollip is able to interact with phosphoinositides, thus participating in membrane trafficking [75]. Tollip is also thought to participate in trafficking of ubiquitinated proteins through interactions with its “coupling of ubiquitin conjugation to endoplasmic reticulum degradation” (CUE) domain [76]. Accordingly, Tollip was localized in senile plaques in Alzheimer’s disease brain [77], which has been associated with neuronal intranuclear inclusions in a mouse model of Huntington disease [78] and prevented toxicity of polyglutamine repeats in cultured cells [79, 80]. Thus, Tollip, through its ubiquitin-binding CUE domain, is proposed to have a critical function in autophagic clearance of protein aggregates. Finally, two studies suggest a direct link between Tollip and mitochondria [34, 81]. Interestingly, ubiquitination abnormality, protein aggregates, and mitochondrial dysfunctions are cardinal features of the PD pathogenesis [82, 83].

We did not observe an increase in Tollip transcripts after LPS treatment at any dose. This data is consistent with findings of Lo and colleagues who similarly did not detect an increase of the major Tollip transcript after LPS treatment in mouse macrophages [35]. Nevertheless, an increase in Tollip protein abundance has been reported in vivo in murine brain after ischemia/reperfusion injury [33] and in vitro in monocytic cells after LPS challenge [45]. The later authors suggest that LPS-induced post-transcriptional events may contribute to such effect and propose that LPS treatment stabilizes endogenous Tollip protein, rather than promoting a Tollip neo-synthesis.

Our results, which show that the lack of Tollip aggravates the inflammatory response elicited by cerebral infusion of LPS, constitute a first evidence for a role of Tollip in the early phase of neuroinflammation. As chronic inflammation has been implicated in PD pathogenesis, the impact of Tollip deletion at later time points after LPS administration remains to be evaluated.

## Conclusions

Several pioneers’ studies have described Tollip as a modulator of the TLR4 and IL-1β pathway in immune cells, exerting an inhibitory action on NF-κB activation. The results of our study show that Tollip deletion exacerbated iNOS production, protein nitration, and DNA oxidation induced by LPS injection in the substantia nigra, a structure affected in PD. These findings highlight a critical role for Tollip in the early phase of TLR4-mediated neuroinflammation. As brain inflammation is known to contribute to PD, Tollip may be a potential target to provide a neuroprotective effect by reducing neuroinflammation. Surprisingly, a high abundance of Tollip protein in dopaminergic neurons is herein described for the first time. This high expression level may reflect a tighter control of the NF-κB signal transduction cascade in the substantia nigra, which is particularly vulnerable to neuroinflammation and oxidative stress. A potential role for Tollip as a target for PD drug development remains to be investigated.

## Additional files

Additional file 1: Figure S2. No alteration of Tollip expression 6 h after intra-nigral LPS injection. A. Up - position of the analyzed sections in the Paxinos Atlas [84]. Down - representative Tollip (in green) and tyrosine hydroxylase (TH) (in red) immunostainings and DAPI staining (in blue) in the SNc. Magnification = ×20. Scale bar = 50 μm. B. Up - figure extracted from Paxinos rat brain atlas showing the part of midbrain extracted for all qPCR experiments. Down - Tollip mRNA amounts in midbrain extracts normalized by β-actin and GAPDH. WT mice underwent PBS or LPS (0.01 or 0.1 or 1 μg) injection (*n* = 3 WT mice per group). Results are expressed as mean ± SEM. (TIF 6918 kb)
Additional file 2: Figure S1. No residual Tollip immunoreactivity in Tollip KO mice. Representative Tollip (in green) and tyrosine hydroxylase (TH) (in red), immunostainings and DAPI staining (in blue) in the SNc of non-injected Tollip WT (panels A and B) and Tollip KO (panel C) mice. Merged - co-localization of Tollip, TH, and DAPI. A. Magnification = ×20. Scale bar = 50 μm. Insets show the region photographed in panel B. B–C Magnification = ×63. Scale bar = 50 μm. Arrows indicate Tollip-positive cells which are not co-labeled with TH. (TIF 15231 kb)
Additional file 3: Figure S3. LPS induces a dose-dependent increase of the pro-inflammatory cytokines IL-1β, IL-6, and TNF-α 6 h after injection. WT mice underwent PBS or LPS (0.01 or 0.1 or 1 μg) injection (*n* = 3 mice per group). mRNA amounts in midbrain extracts are normalized by β-actin and GAPDH. Results are expressed as mean ± SEM, **P* < 0.05 versus PBS group, using a Kruskal–Wallis test followed by a Dunn’s post hoc test. (TIF 1059 kb)
Additional file 4: Figure S4. No induction of iNOS 6 h after PBS injection in the midbrain of Tollip KO mice. A representative iNOS and NeuN immunostaining in the midbrain in contralateral and ipsilateral side of the PBS (1 μL) injection, in Tollip KO mice. Magnification = ×40. Scale bar = 30 μm. (TIF 740 kb)
Additional file 5: Figure S5. LPS at three doses did not increase CD68 mRNA into the midbrain of WT mice, at 6-h post-injection. WT mice underwent intra-nigral PBS or LPS (0.01 or 0.1 or 1 μg) injection (*n* = 3 WT mice per group). mRNA amounts in midbrain extracts are normalized by β-actin and GAPDH. Results are expressed as mean ± SEM. (TIF 327 kb)

## Abbreviations

8-oxo-dG: 8-oxo-2′-deoxyguanosine
DAPI: 4′,6-diamidino-2-phenylindole
eGFP: Enhanced green fluorescent protein
ELISA: Enzyme-linked immunosorbent assay
GAPDH: Glyceraldehyde 3-phosphate dehydrogenase
GFAP: Glial fibrillary acidic protein
IFN-γ: Interferon gamma
IL-1β: Interleukin 1 beta
IL-6: Interleukin 6
iNOS: Inducible nitric oxide synthase
KO: Knockout
LPS: Lipopolysaccharide
NeuN: Neuronal nuclei
NF-κB: Nuclear factor kappa B
NFH: Neurofilament H
NO: Nitric oxide
PD: Parkinson’s disease
rAAV: Recombinant adeno-associated virus
SNc: Substantia nigra pars compacta
TH: Tyrosine hydroxylase
TLR4: Toll-like receptor 4
TNF-α: Tumor necrosis factor alpha
Tollip: Toll-like interacting protein
WT: Wild-type
