# Supplementary figures and images for: Tollip, an early regulator of the acute inflammatory response in the substantia nigra
Source: J Neuroinflammation. 2016 Dec 7;13:303. doi: 10.1186/s12974-016-0766-5 (PMC5142340; doi:10.1186/s12974-016-0766-5)

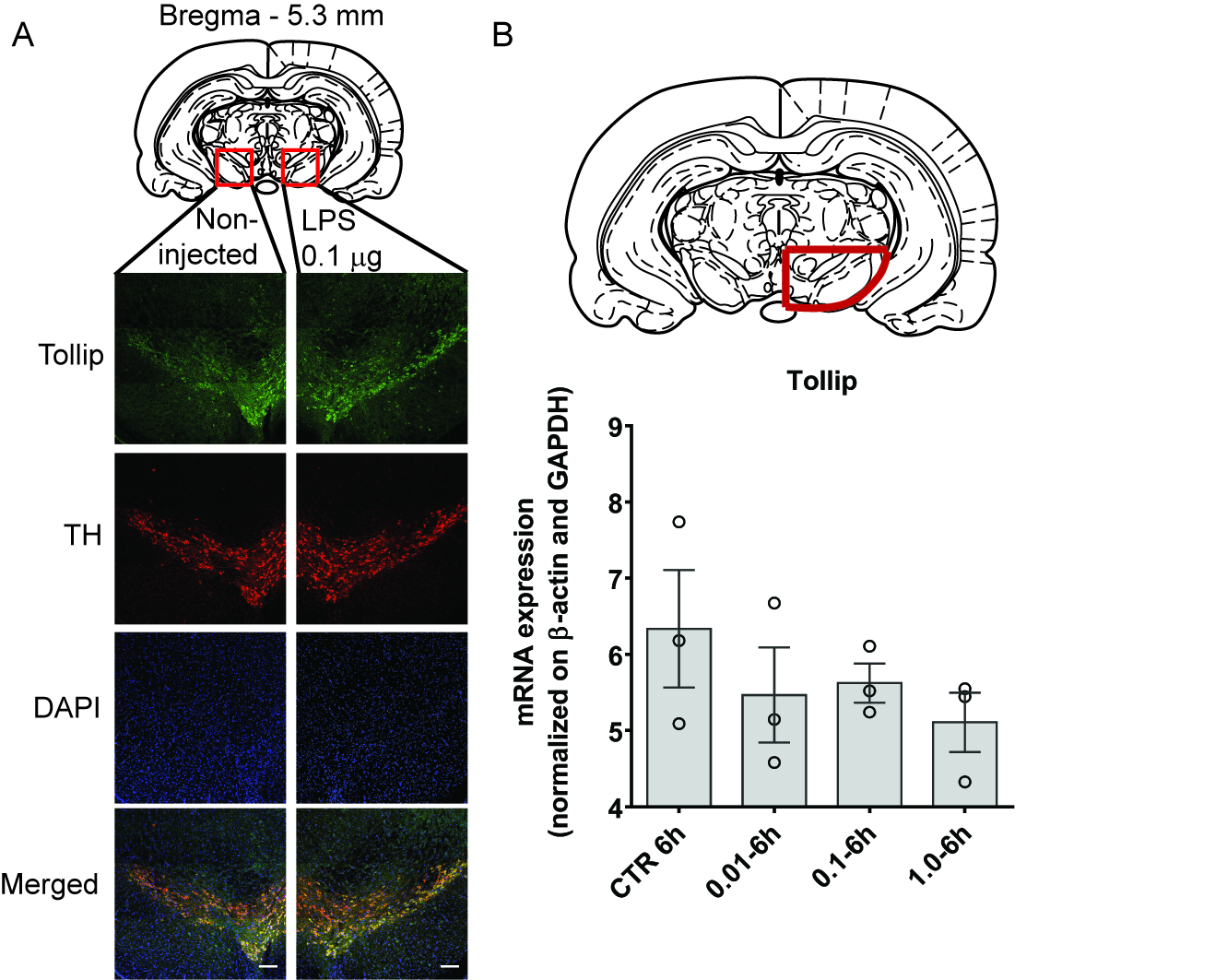

Supplement: Additional file 1: Figure S2. — No alteration of Tollip expression 6 h after intra-nigral LPS injection. A. Up - position of the analyzed sections in the Paxinos Atlas [84]. Down - representative Tollip (in green) and tyrosine hydroxylase (TH) (in red) immunostainings and DAPI staining (in blue) in the SNc. Magnification = ×20. Scale bar = 50 μm. B. Up - figure extracted from Paxinos rat brain atlas showing the part of midbrain extracted for all qPCR experiments. Down - Tollip mRNA amounts in midbrain extracts normalized by β-actin and GAPDH. WT mice underwent PBS or LPS (0.01 or 0.1 or 1 μg) injection (n = 3 WT mice per group). Results are expressed as mean ± SEM. (TIF 6918 kb) [file 12974_2016_766_MOESM1_ESM.tif]

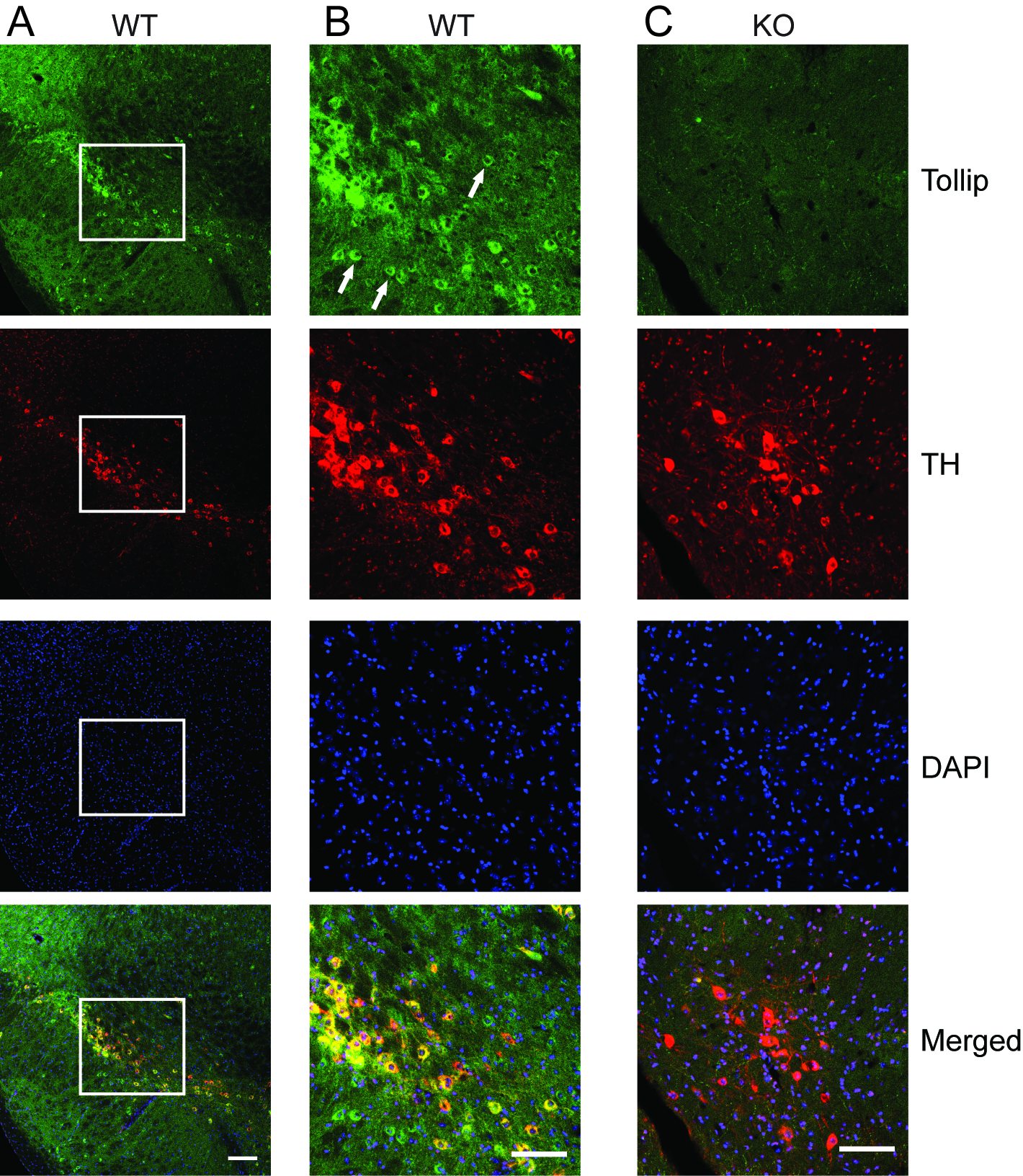

Supplement: Additional file 2: Figure S1. — No residual Tollip immunoreactivity in Tollip KO mice. Representative Tollip (in green) and tyrosine hydroxylase (TH) (in red), immunostainings and DAPI staining (in blue) in the SNc of non-injected Tollip WT (panels A and B) and Tollip KO (panel C) mice. Merged - co-localization of Tollip, TH, and DAPI. A. Magnification = ×20. Scale bar = 50 μm. Insets show the region photographed in panel B. B–C Magnification = ×63. Scale bar = 50 μm. Arrows indicate Tollip-positive cells which are not co-labeled with TH. (TIF 15231 kb) [file 12974_2016_766_MOESM2_ESM.tif]

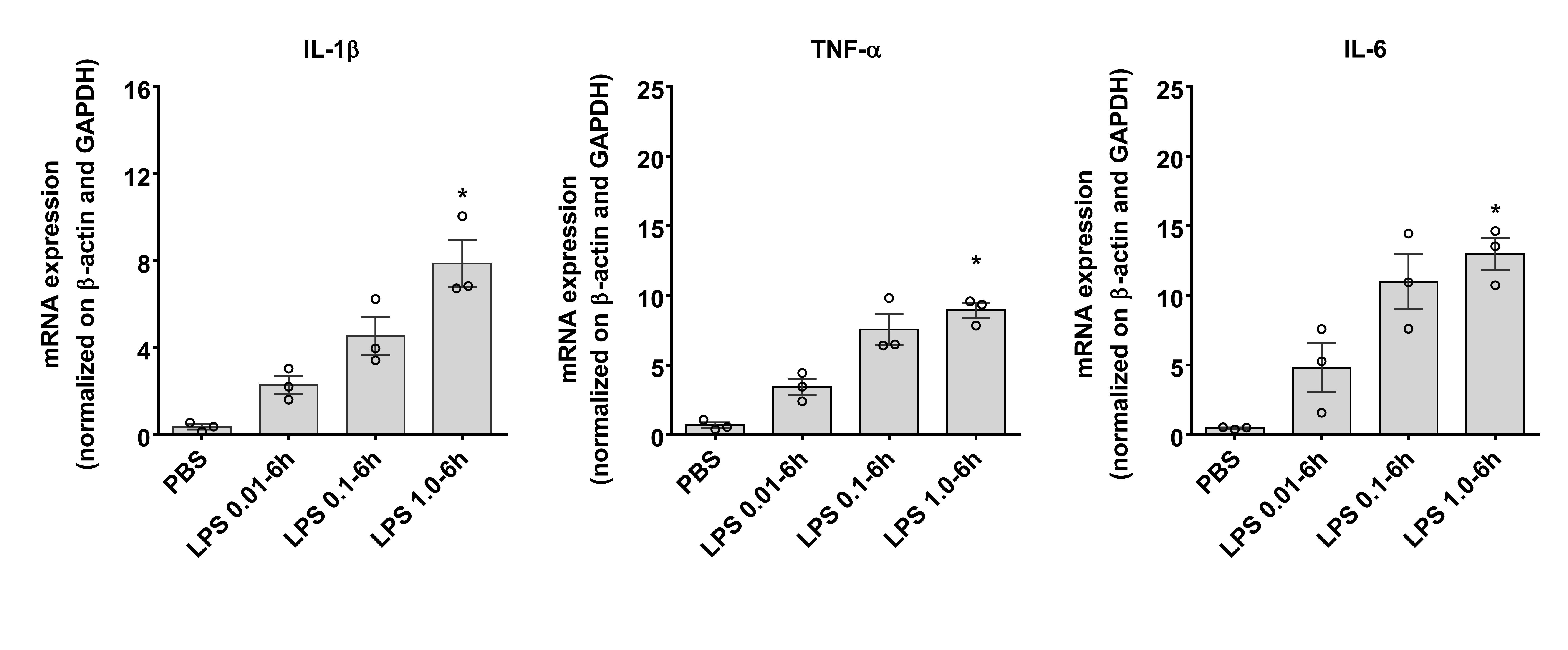

Supplement: Additional file 3: Figure S3. — LPS induces a dose-dependent increase of the pro-inflammatory cytokines IL-1β, IL-6, and TNF-α 6 h after injection. WT mice underwent PBS or LPS (0.01 or 0.1 or 1 μg) injection (n = 3 mice per group). mRNA amounts in midbrain extracts are normalized by β-actin and GAPDH. Results are expressed as mean ± SEM, *P < 0.05 versus PBS group, using a Kruskal–Wallis test followed by a Dunn’s post hoc test. (TIF 1059 kb) [file 12974_2016_766_MOESM3_ESM.tif]

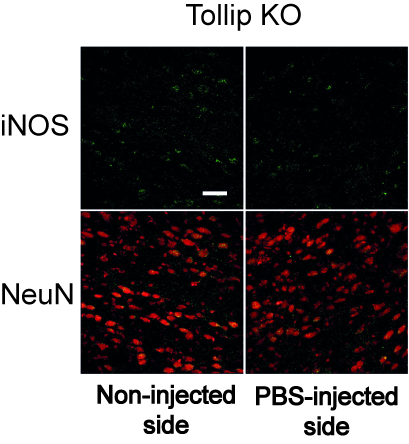

Supplement: Additional file 4: Figure S4. — No induction of iNOS 6 h after PBS injection in the midbrain of Tollip KO mice. A representative iNOS and NeuN immunostaining in the midbrain in contralateral and ipsilateral side of the PBS (1 μL) injection, in Tollip KO mice. Magnification = ×40. Scale bar = 30 μm. (TIF 740 kb) [file 12974_2016_766_MOESM4_ESM.tif]

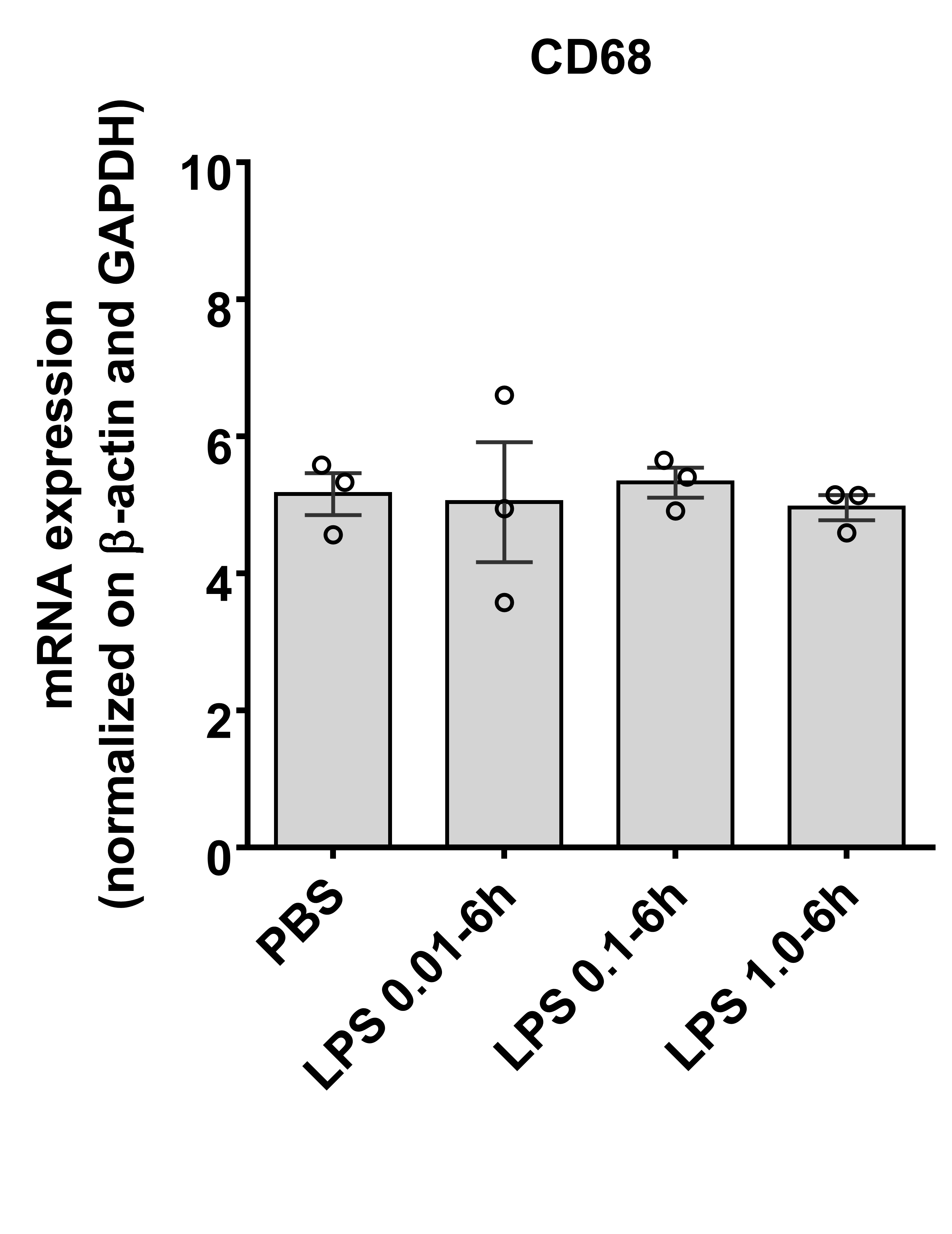

Supplement: Additional file 5: Figure S5. — LPS at three doses did not increase CD68 mRNA into the midbrain of WT mice, at 6-h post-injection. WT mice underwent intra-nigral PBS or LPS (0.01 or 0.1 or 1 μg) injection (n = 3 WT mice per group). mRNA amounts in midbrain extracts are normalized by β-actin and GAPDH. Results are expressed as mean ± SEM. (TIF 327 kb) [file 12974_2016_766_MOESM5_ESM.tif]
